# Editorial: Further advances in understanding the endocrine cancer microenvironment

**DOI:** 10.3389/fendo.2022.1009963

**Published:** 2022-08-24

**Authors:** Francesca Coperchini, Rosa Marina Melillo, Mario Rotondi

**Affiliations:** ^1^ Istituti Clinici Scientifici Maugeri IRCCS, Unit of Internal Medicine and Endocrinology, Laboratory for Endocrine Disruptors, Pavia, Italy; ^2^ Department of Molecular Medicine and Medical Biotechnology, University of Naples Federico II, Naples, Italy; ^3^ Institute of Experimental Endocrinology and Oncology (IEOS), CNR, Naples, Italy; ^4^ Department of Internal Medicine and Therapeutics, University of Pavia, Pavia, Italy

**Keywords:** tumor microenvironment, endocrine cancer, CXCL8 (Interleukin-8), endocrine tumor cells, immunotherapy

Inflammation is a physiologic process occurring in response to tissue damage. Already in 1863, Virchow (1, 2), based on the observation that leukocytes infiltrate neoplastic tissues, hypothesized a relationship between inflammation and cancer. The demonstration that inflammation promotes tumor genome instability, cell growth, survival, invasion and angiogenesis has led to the current notion that inflammation is an essential component of malignancies (1) suggesting that it could represent a target for cancer therapy.

More recently, the term tumor microenvironment (TME) was used to include both cellular and soluble components which surround and infiltrate the tumor mass (3). The TME is composed of extracellular matrix and stromal cells, including fibroblasts, vessel cells (endothelial cells, pericytes, and smooth muscle cells), and inflammatory leukocytes (lymphocytes, macrophages, dendritic cells, mast cells, and neutrophils), while soluble mediators include a wide spectrum of chemokines, cytokines, and growth factors, which are secreted by both resident tumor and surrounding normal cells as well as by infiltrating immune cells (4–6).

It progressively became clear that both the phenotype and the number of infiltrating cells are strongly dependent upon specific chemokines secreted within the TME. Thus, chemokines rapidly became among the most extensively characterized molecules involved in the maintenance and progression of tumor-related inflammation (7).

In the five articles included in this Research Topic, different aspects of the most recent lines of research on the field of TME and cancer biology were addressed. The findings are here briefly overviewed with the final aim to provide a stimulating summary of the present knowledge.

## Specific gene expression by tumor cells and the TME

Huang et al, by performing a Gene-Set-Enrichment-Analysis on a large group of patients with breast cancer, demonstrated that the expression levels of ITK gene (among several other genes) were significantly and positively related to prolonged overall survival. Given that the IL-2-inducible T-cell kinase (ITK) gene regulates T cell signaling and subsequent secretion of several pro-inflammatory cytokines (8), it is directly involved in determining the proportion and the phenotype of tumor-infiltrating immune cells. Thus, the results would confirm that the immune components in TME contribute to the prognosis of patients and suggest that TME-related genes may become a therapeutic target in clinical practice.

By using a similar approach (GSEA), Qiu et al. analyzed NBPF1 (a member of the neuroblastoma breakpoint family) (9) gene expression in cancer and investigated the relationship between NBPF1, TME and prognostic outcome in patients with adrenocortical carcinoma (ACC). They found that NBPF1 was an independent prognostic factor for ACC, and that NBPF1 expression was inversely correlated with immune cell infiltration in several tumors. Furthermore, they suggest that NBPF1 may act as an anti-inflammatory factor in the anti-tumor immune response of ACC. The results indicate that NBPF1 could serve as a prognostic biomarker for multiple cancers, being a key player in the tumor immune microenvironment composition and in tumor immunity, which would ultimately suggest that anti-NBPF1 immunotherapy might be useful for ACC treatment.

## Soluble mediators in the TME

Cell adhesion molecules (CAMs) are transmembrane receptor proteins involved in cell-to-cell or cell-to-extracellular matrix binding (10). CAMs are responsible for the interplay between cancer cells and the TME ultimately promoting drug resistance, which in turn leads to therapeutic failure of chemotherapy (11, 12). Thus, CAMs could represent potential therapeutic targets for cancer intervention. Based on these notions, Ruan et al., aimed at reviewing most recent evidences on the role of integrins, cadherins, selectins, and CD44 in endocrine-related cancer. Briefly, an overview of the mechanisms by which altered expression of CAMs lead to remodeling of TME and subsequent development of drug resistance of cancer cells is provided, and their application also to endocrine-related cancer is discussed.

By going further, IGFs are abundantly secreted within the TME, where they enhance angiogenesis and promote the maintenance, proliferation, and migration of cancer cells. IGFs also play a major role in the onset of acquired chemoresistance in several tumor cells (13). Thus, anti-IGF therapies emerged as a promising strategy against cancer, especially for overcoming cancer drug resistance both in *in vivo* and *in vitro* preclinical studies. However, high toxicity and resistance characterize these agents as reported by clinical trials (14).

Kamdje et al. reviewed data supporting that TME exerts pro-tumorigenic effects, which are at least in part, dependent upon the IGF-1 signaling pathway. More interestingly, the therapeutic potential of targeting the IGF-1 receptor to overcome resistance to chemotherapy drugs in endocrine-related cancer was discussed.

Several lines of evidence indicated that reducing CCL2 and CXCL8 (chemokines with well-established pro-tumorigenic effects) secretion within the TME provides beneficial effects in terms of slowing down tumor progression. Vitamin D displays several *in vitro* anti-cancer effects, which include anti-proliferative, antiangiogenetic, anti-metastatic, pro-differentiating, and pro-apoptotic properties in several types of cancer cells (15). Furthermore, high serum vitamin D levels were reported to be associated with lower cancer incidence in humans (15, 16). Coperchini et al, evaluated whether Vitamin D could modulate CXC8 and CCL2 secretion by thyroid tumor cells, ultimately reducing their migration. The results showed that vitamin D treatment inhibited the secretion of CCL2 but not of CXCL8, while independently reduced thyroid cancer cell migration.

In conclusion, although a limited number of studies were collected in the present Research Topic, it is clear that the Endocrine Cancer Microenvironment represents a major target for developing novel anti-cancer strategies. Currently available evidences have consistently supported the concept that cancer progression is promoted by the complex interplay between malignant cells and the surrounding environment (17–19). Tumor related inflammation represents the common denominator but genetic aspects, specific cellular types and soluble molecules within the TME may all represent potential reliable models for future research.

## Author contributions

All authors listed have made a substantial, direct, and intellectual contribution to the work and approved it for publication.

## Conflict of interest

The authors declare that the research was conducted in the absence of any commercial or financial relationships that could be construed as a potential conflict of interest.

## Publisher’s note

All claims expressed in this article are solely those of the authors and do not necessarily represent those of their affiliated organizations, or those of the publisher, the editors and the reviewers. Any product that may be evaluated in this article, or claim that may be made by its manufacturer, is not guaranteed or endorsed by the publisher.
